# lncRNA-miRNA-mRNA network in kidney transcriptome of *Labeo rohita* under hypersaline environment

**DOI:** 10.1038/s41597-024-03056-y

**Published:** 2024-02-22

**Authors:** Nitin Shukla, Vemula Harshini, Ishan Raval, Amrutlal K. Patel, Chaitanya G. Joshi

**Affiliations:** Gujarat Biotechnology Research Centre, Sector 11, Gandhinagar, Gujarat India

**Keywords:** Gene regulatory networks, Transcriptomics

## Abstract

The present study describes the kidney transcriptome of *Labeo rohita*, a freshwater fish, exposed to gradually increased salinity concentrations (2, 4, 6 and 8ppt). A total of 10.25 Gbps data was generated, and a suite of bioinformatics tools, including FEELnc, CPC2 and BLASTn were employed for identification of long non-coding RNAs (lncRNAs) and micro RNAs (miRNAs). Our analysis revealed a total of 170, 118, 99, and 269 differentially expressed lncRNA and 120, 118, 99, and 124 differentially expressed miRNAs in 2, 4, 6 and 8 ppt treatment groups respectively. Two competing endogenous RNA (ceRNA) networks were constructed i.e. A* ceRNA network with up-regulated lncRNAs and mRNAs, down-regulated miRNAs; and B* ceRNA network vice versa. 2ppt group had 131 and 83 lncRNA-miRNA-mRNA pairs in A* and B* networks, respectively. 4ppt group featured 163 pairs in A* network and 191 in B* network, while the 6ppt had 103 and 105 pairs. 8ppt group included 192 and 174 pairs. These networks illuminate the intricate RNA interactions in freshwater fish to varying salinity conditions.

## Background & Summary

Osmoregulation is a crucial mechanism in fishes to adapt against acute or chronic changes in environmental salinity. Kidney is one of the crucial osmoregulatory organs in fishes to maintain an osmotic balance of body fluids through water influx or efflux^1^. In freshwater fishes, kidney excrete large volumes of hypotonic urine and reabsorb active ions to maintain ionic homeostasis^2^. Among three major carp species in India, *Labeo rohita* (rohu) is the most important freshwater fish. The species have higher consumer demand and economic value^3^. In recent years, the impact of climate change causes an increase in salinity levels in freshwater resources^4^, significantly impacting aquatic organisms’ physiology^5,6^. In a salinity-fluctuating environment, maintaining internal osmotic and ionic homeostasis and adapting to salinity changes involves participating in various enzymes and transporters^7,8^. The first step toward elucidating molecular mechanisms and core physiological processes behind salinity change is identifying the candidate genes involved^9^.

The non-coding RNAs, such as miRNAs and lncRNAs, are reported to be regulators of mRNAs at the transcriptional and post-transcriptional levels^10,11^. The competitive endogenous (ceRNA) hypothesis demonstrates that lncRNAs can act as endogenous sponges to regulate mRNAs expression by negatively mediating miRNAs expression^12,13^. There were previous reports focused on role of miRNAs in the regulation of osmotic pressure^14^, salinity stress^15^, and immune response^16^, also studies on lncRNA regulation of mRNAs under adverse environmental conditions^17^. In order to find possible immune response regulators that could be challenged by the pathogenic bacterium *Aeromonas salmonicida*, ceRNA analysis was conducted in Atlantic salmon^16^.

**Fig. 1:**
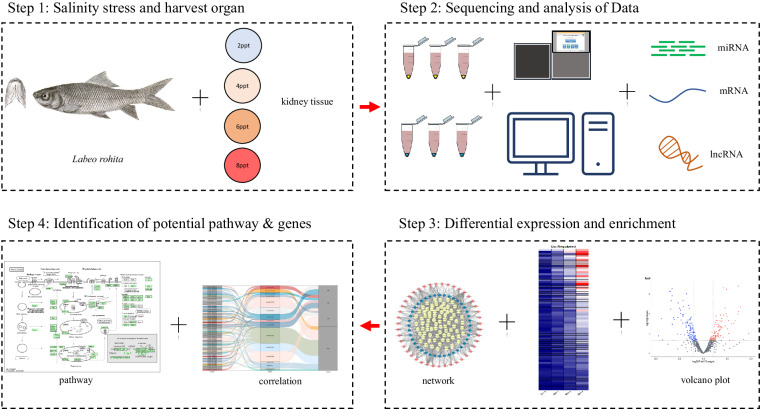
Study design of the kidney transcriptome profile under hypersaline environment.

In the present study, *L. rohita* was treated with 2, 4, 6 and 8ppt salinity concentration and kidney tissue samples were processed for transcriptome sequencing. Differentially expressed mRNAs, miRNAs and lncRNAs were identified. Based on target prediction and correlation analysis ceRNA network was generated. This data will be helpful to the research community in understanding the physiology of fish in hypersaline conditions. The schematic representation of study design and workflow is presented in Fig. 1.

## Methods and Results

### Ethical approval

All the experimental protocols were approved by Institute biosafety committee of PGIFER (Postgraduate Institute of Fisheries Education and Research), Kamdhenu University, Gandhinagar, Gujarat. The guidelines of the CPCSEA (Committee for the Purpose of Control and Supervision of Experiments on Animals, Ministry of Environment and Forests (Animal Welfare Division) on care and use of animals and ARRIVE2.0 (Animal Research: Reporting of *In Vivo* Experiments) in scientific research were followed during the experiment.

### Sample collection and library preparation

The salinity stress experiment was conducted at Postgraduate Institute of Fisheries Education and Research (PGIFER), Kamdhenu University, Himmatnagar, Gujarat. Fingerlings (>10 g) were acquired from the State Fisheries Department Fish Hatchery, Gujarat. They were kept in 150-liter tanks with continuous aeration at 27 ± 5 °C. The fish were fed at 5% of the body weight till the end of the experiment, and 25% water was replaced each day, along with feces, to keep the tanks clean. The fingerlings were randomly split into control and salinity treatment groups. The control group was constantly maintained at 0ppt whereas in the treatment group the salinity was gradually raised (1ppt/day) to 2, 4, 6 and 8 ppt salinity by adding (55 ppt) of Red Sea Coral Pro Salt (Red Sea, USA). Each week the fish were gradually transferred to increased salinity and 3 fish were randomly euthanized and tissue samples were collected from the control and treatment groups. The samples were stored at −80 °C in RNAlater^®^ until further use. The total RNA was extracted from the kidney tissues using RNeasy Plus Mini Kit (Qiagen, Germany). The integrity and quality of RNA were assessed with Agilent 2100 Bioanalyzer system (Agilent technologies, Ca) and Qubit 4 Fluorometer (Thermo Fisher Scientific, United States). The cDNA libraries were prepared by TruSeq Stranded Total RNA Library Prep Kit (Illumina, Ca) after removing ribosomal RNA with RiboMinus™ Eukaryote System v2 (Thermo Fisher, Ma). The samples were sequenced on an Illumina MiSeq and NovaSeq 6000 platform with paired-end forward and reverse reads.

### Data processing and expression analysis

A total of 10.25 Gbps data was generated and processed for a quality check using FastQC (v0.11.9). The reads were aligned with the NCBI reference genome Rohu *(Labeo rohita*) (GenBank assembly accession GCA_004120215.1 v1) using segemehl (v0.2.0-418), and expression levels of mRNAs were computed with featureCounts (v2.0.1). The expression matrix of mRNA genes from individual salinities was used in the DESeq 2 package for differential expression analysis. The significant DEGs were considered with p-value ≤ 0.05 | log2FoldChange ≥0.5 for the enrichment and pathway analysis. The data was visualized using the ggplot2 package for each salinity-treated group. The detailed results of expression profile of transcriptome of kidney and significant mRNAs can be found in our previously published study^18^.

### Prediction of putative lncRNAs

**Fig. 2:**
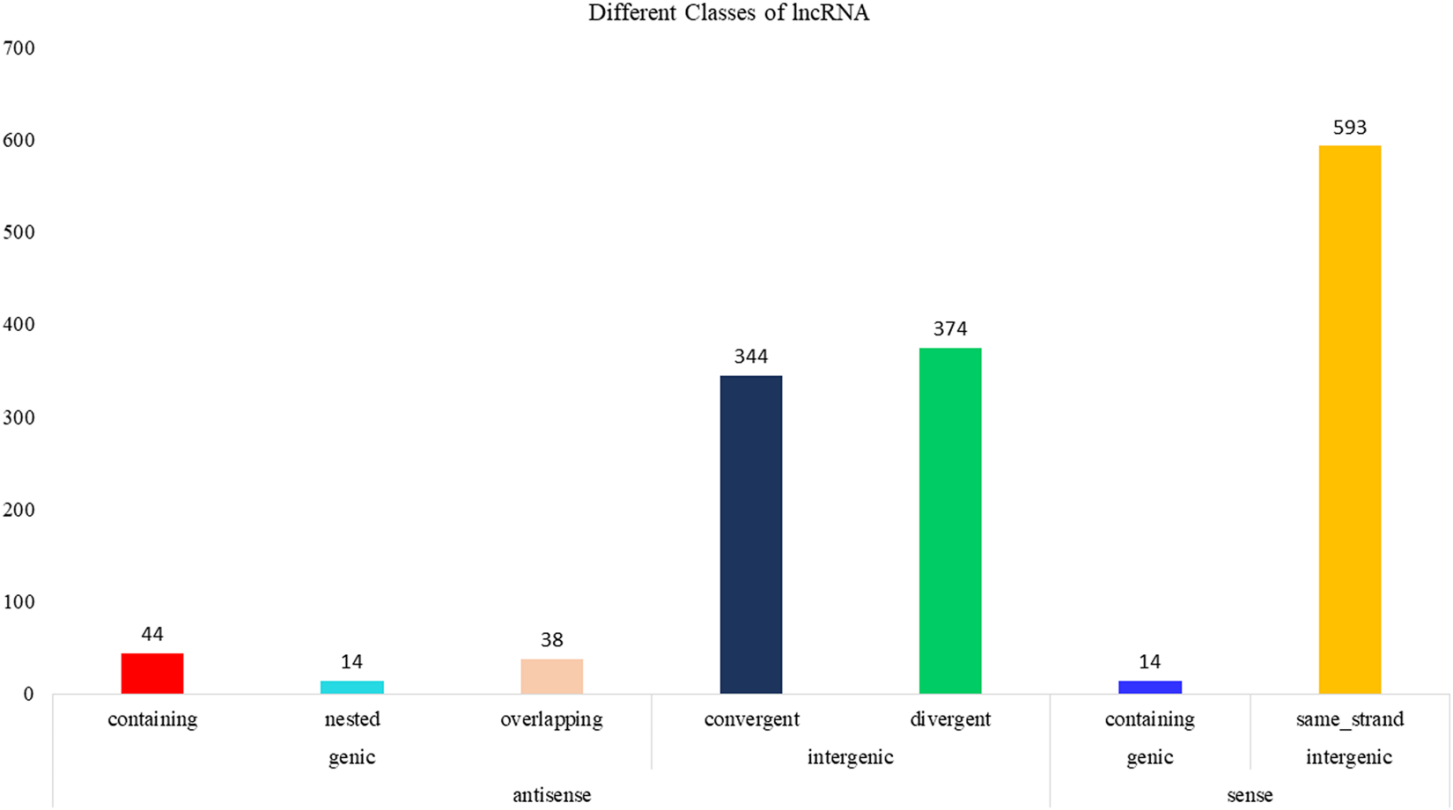
Bar plot representing various classes of lncRNAs predicted in kidney transcriptome of *Labeo rohita*.

**Fig. 3:**
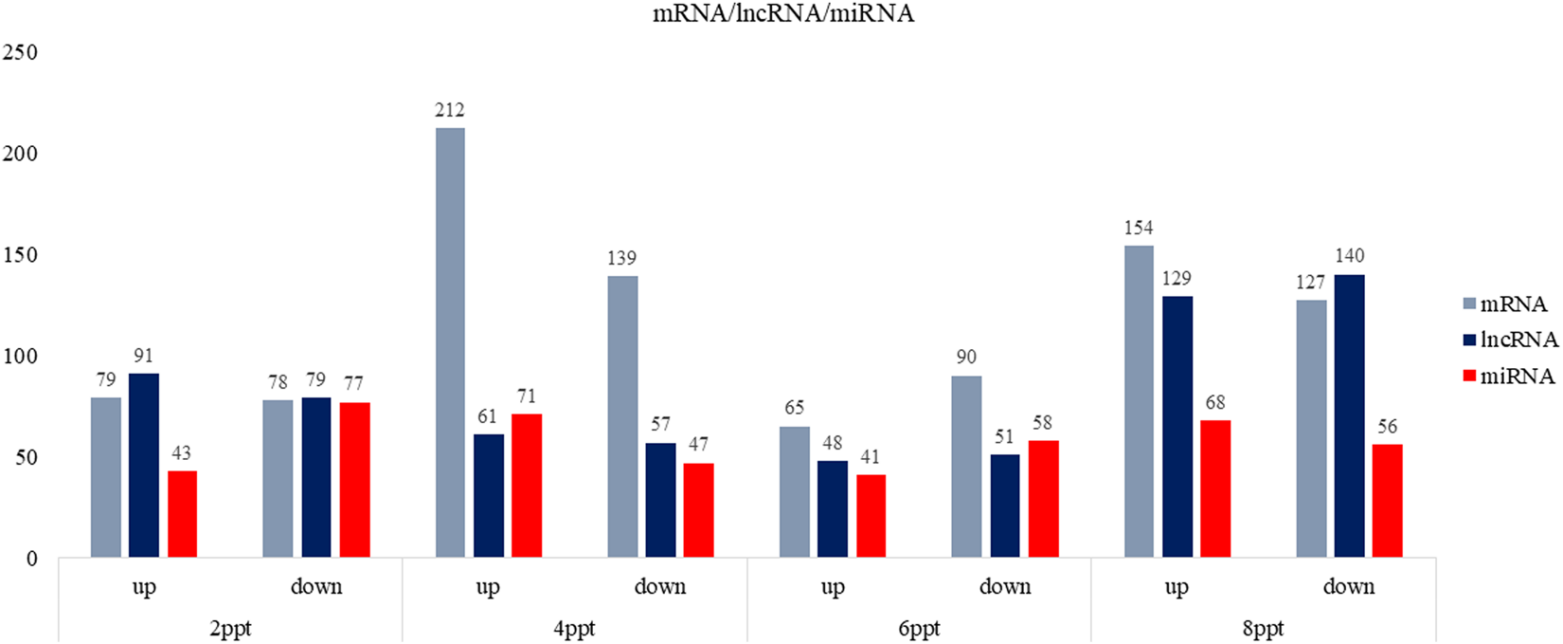
Bar plot depicting no. of differentially expressed mRNAs, lncRNAs and miRNAs identified in 2, 4, 6, and 8ppt salinity treated groups.

**Fig. 4:**
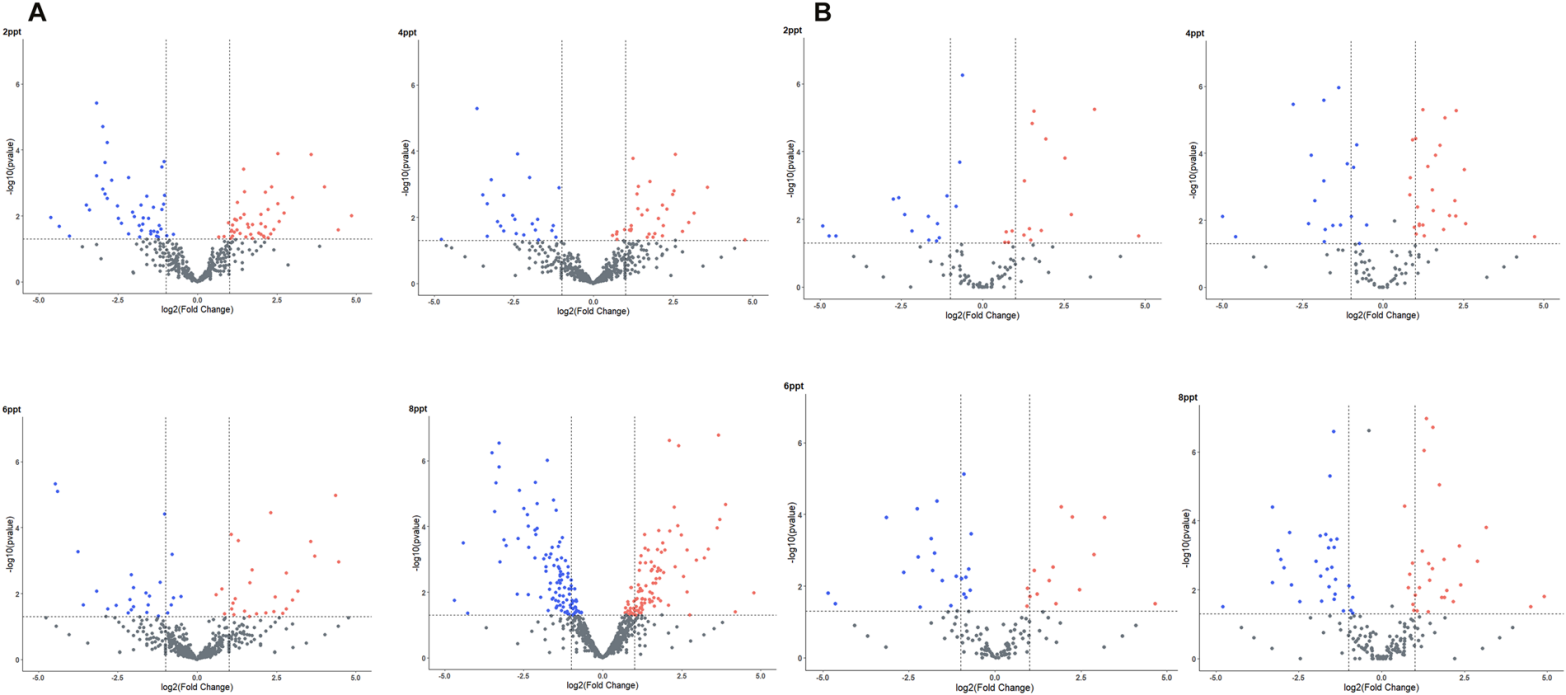
Volcano plot representing differentially expressed (**A**) long non-coding RNAs (lncRNAs) (**B**) micro RNAs (miRNAs) at 2, 4, 6, and 8ppt salinity treated groups. The red and blue colour indicates up and down regulated genes respectively, whereas the grey colour indicates non-significant genes.

For identification of lncRNAs, transcripts were de-novo assembled with Cufflinks version (v2.2.1) using aligned bam files from individual samples. Cuffmerge was used to obtain a combined assembly, which was then processed through FEELnc pipeline (v.0.2.1) (https://github.com/tderrien/FEELnc)^19^. FEELnc_filter_ was initially utilized to filter out transcripts less than 200 bp, including single-exon transcripts. Next, FEELnc_codpot_ was used to evaluate the coding potential of each transcript based on the length of ORF, sequence bias, and transcript length to differentiate lncRNA from mRNA. Of 37,462 transcripts, 4,170 potential candidate lncRNAs were identified from the FEELnc program. Subsequently, FEELnc_classifier_ was used to classify the identified lncRNA into genic, intergenic, containing, same strand, convergent, divergent, overlapping, and nested categories (Fig. 2). Finally, CPC2 (v0.1) (http://cpc2.gao-lab.org/) was utilized as an additional assessment method for the identification of the coding potential of transcripts, which uses a support vector machine (svm)^20^, 1,447 non-coding lncRNA were finalized, and an input matrix was prepared with featureCounts using GTF file of lncRNA for differential expression analysis using DESeq2 package in R software (v 4.2.3). In the 2, 4, 6, and 8ppt salinity groups, 170, 118, 99, and 269 differentially expressed lncRNA with p-value ≤ 0.05 & Log2FoldChange ≥0.5, respectively (Figs. 3, 4 and Figshare Dataset 1^21^).

### Prediction of putative miRNAs

To identify miRNAs, fasta file was prepared from raw fastq using the fastx toolkit (https://github.com/agordon/fastx_toolkit). The collapsed reads function from the mirdeep2 package was implemented to identify miRNA sequence whose length varies between (16 to 24 bp) which is shorter than the sequence read length. The standalone BLASTn tool was implemented for the identification of putative mature miRNA sequences obtained from the miRbase database (https://www.mirbase.org), for teleostei species with E-value (1E-1) and percent identity ≥ 95 as a cut-off. The Differential expression analysis of miRNAs was performed using EdgeR package from Bioconductor (v.3.40.2)^22^. A total of 120, 118, 99, and 124 differentially expressed miRNAs with p-value ≤ 0.05 and log2FoldChange ≥0.5 were considered (Figs. 3, 4 and Figshare Dataset 2^21^).

### Identification of target mRNAs for lncRNA and miRNA

LncRNAs competitively bind microRNAs to alter the expression of specific mRNAs^23^. The targeted mRNAs were predicted for miRNAs and lncRNAs using miRanda (v3.3a) (http://www.microrna.org/microrna/home.do)^24^, which uses scoring matrix for the individual alignment for detection of potential target sites in coding sequences, with score cutoff ≥ 145 and energy ≤ −10^25^ to predict lncRNA-miRNA pairs and miRNA-lncRNA pairs. A total of 953, 863, 494 and 1983 lncRNA-miRNA pairs and 766, 869, 532, and 1226 miRNA-mRNA pairs were identified in 2, 4, 6, and 8ppt salinity treated groups, respectively (Figshare Dataset 3^21^). Correlation between lncRNA and miRNA was calculated using corr.test() function by R software. LncRNA-miRNA pairs using Pearson correlation coefficients (PCC) with | r | ≥ 0.94 and p-value ≤ 0.05 were selected. A total of 10,999; 20,341; 7,575; 36,919 significant lncRNA-mRNA pairs were identified in 2, 4, 6, and 8ppt groups respectively. These lncRNA-mRNA pairs include 159 lncRNAs and 152 mRNAs, 118 lncRNAs and 351 mRNAs, 99 lncRNAs and 155 mRNAs, and 268 lncRNAs and 279 mRNAs in 2, 4, 6, and 8ppt groups respectively (Figshare Dataset 4^21^).

### Construction of ceRNA network

**Fig. 5:**
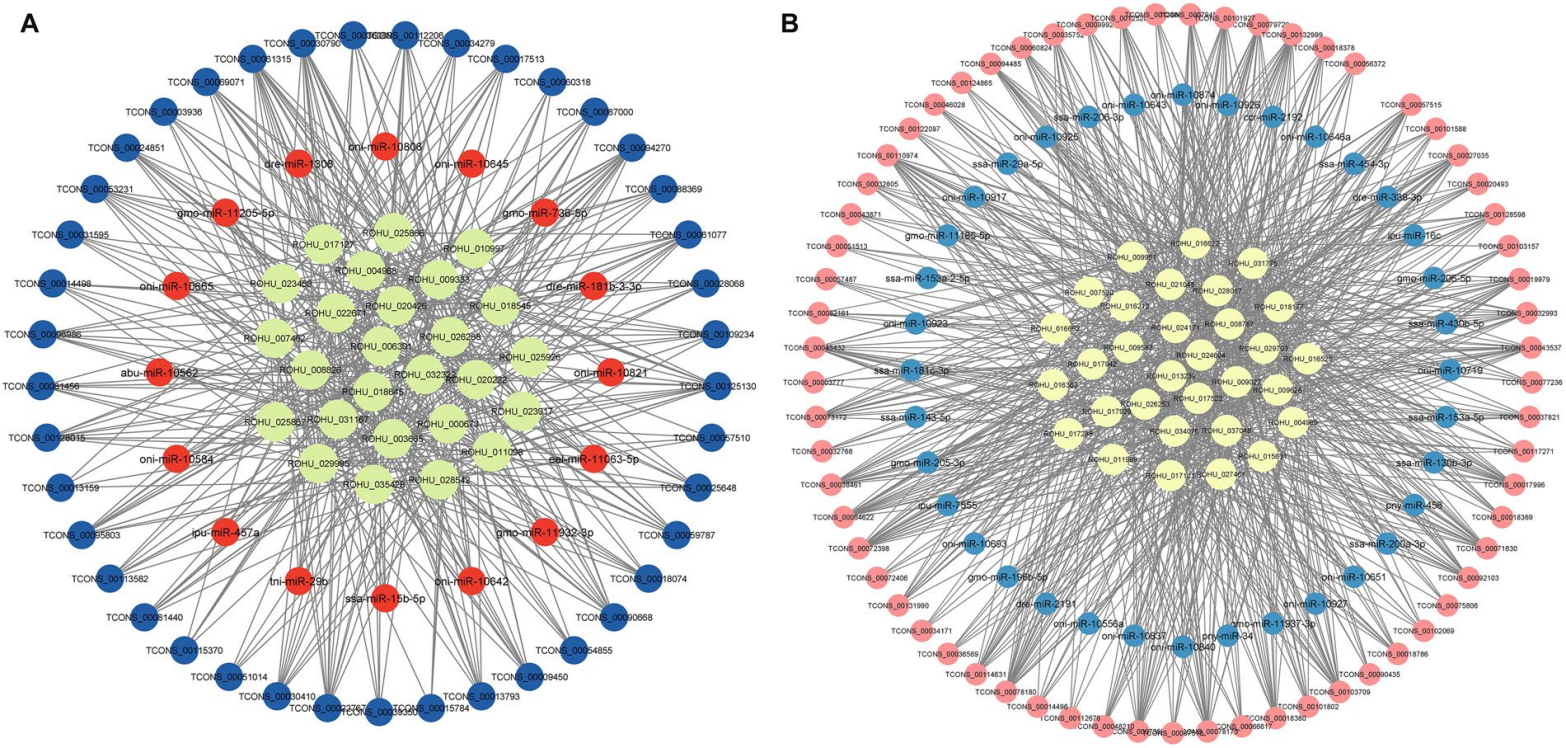
ceRNA (lncRNA-miRNA-mRNA) network of 2ppt salinity treated group. (**A**) The A* ceRNA network. Blue, orange, and green colour circles represent lncRNA, miRNA and mRNA respectively. Network includes 424 edges, and 83 nodes consists of 64, 36 and 31 lncRNAs, miRNAs, and mRNAs respectively. (**B**) The B* ceRNA network. Pink, blue, and yellow colour circles represent lncRNA, miRNA and mRNA respectively. Network includes 880 edges, and 131 nodes consists of 41, 16 and 26 lncRNAs, miRNAs and mRNAs respectively.

Among the predicted, lncRNA-miRNA and miRNA-mRNA pairs, under the stipulation that both lncRNA and mRNA are concurrently targeted by the same miRNA and display a negative co-expression. Those pairs were considered to construct lncRNA-miRNA-mRNA network, and the network topology was graphically depicted using the Cytoscape software (v3.9.1) for visualization and subsequent analysis. According to the ceRNA hypothesis, ceRNAs (lncRNA and mRNA) have positive correlation expression by competing for the same miRNA, which is negatively correlated. Thus, two different ceRNA networks were constructed for each treatment group consisting of, i.e., (1.) up-regulated lncRNAs and mRNAs, and down-regulated miRNAs (A* ceRNA network) and (2.) down-regulated lncRNAs and mRNAs, and up-regulated miRNAs (B* ceRNA network). Both positive and negative correlation pairs were identified based on log2FoldChange values. In the 2ppt treatment group, the A* integrated network contains 131 lncRNA-miRNA-mRNA pairs which include 64 lncRNAs, 36 miRNAs, and 31mRNAs and the B* integrated network contains 83 lncRNA-miRNA-mRNA pairs, including 41 lncRNAs 16 miRNAs and 26mRNAs (Fig. 5 and Figshare Dataset 5^21^).

**Fig. 6:**
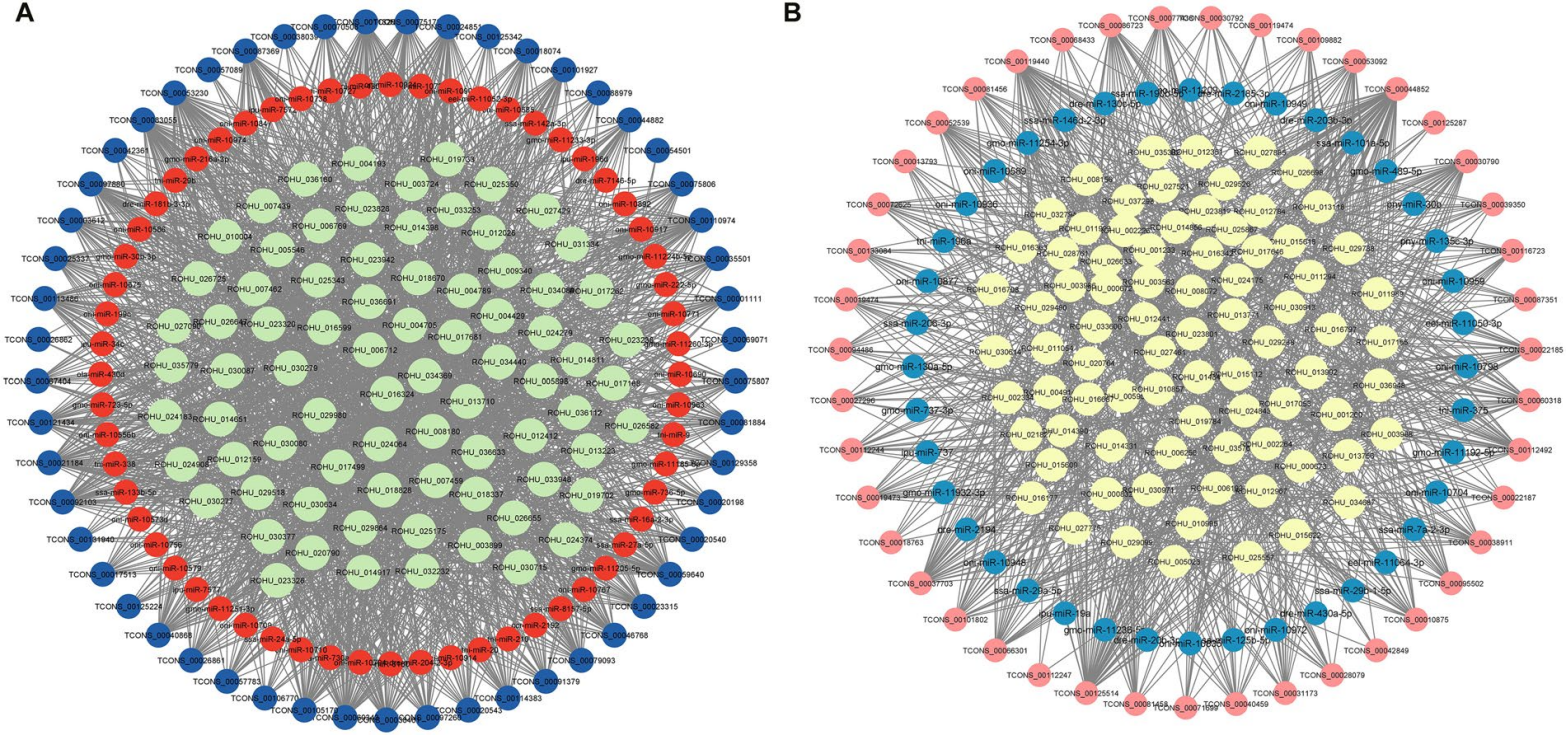
ceRNA (lncRNA-miRNA- mRNA) network of 4ppt salinity treated group. (**A**) The A* ceRNA network. Blue, orange, and green colour circles represent lncRNA, miRNA and mRNA respectively. Network includes 2023 edges, and 191 nodes consists of 43, 40 and 80 lncRNAs, miRNAs, and mRNAs respectively. (**B**) The B* ceRNA network. Pink, blue, and yellow colour circles represent lncRNA, miRNA and mRNA respectively. Network includes 1139 edges, and 163 nodes consists of 53, 60 and 78 lncRNAs, miRNAs and mRNAs respectively.

**Fig. 7:**
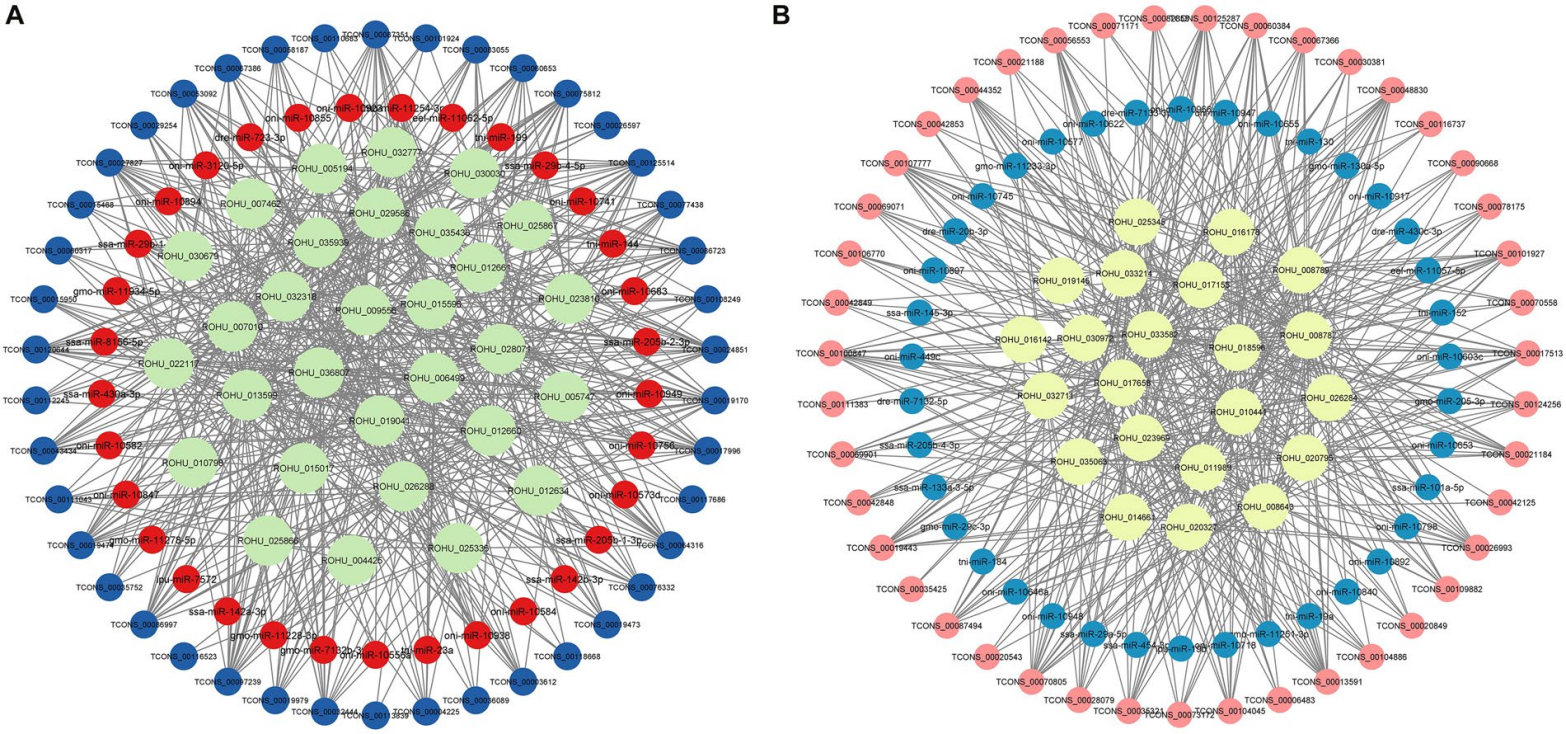
ceRNA (lncRNA-miRNA- mRNA) network of 6ppt salinity treated group. (**A**) The A* ceRNA network. Blue, orange, and green colour circles represent lncRNA, miRNA and mRNA respectively. Network includes 592 edges, and 105 nodes consists of 43, 38 and 22 lncRNAs, miRNAs, and mRNAs respectively. (**B**) The B* ceRNA network. Pink, blue, and yellow colour circles represent lncRNA, miRNA and mRNA respectively. Network includes 477 edges, and 103 nodes consists of 42, 33 and 30 lncRNAs, miRNAs and mRNAs respectively.

**Fig. 8:**
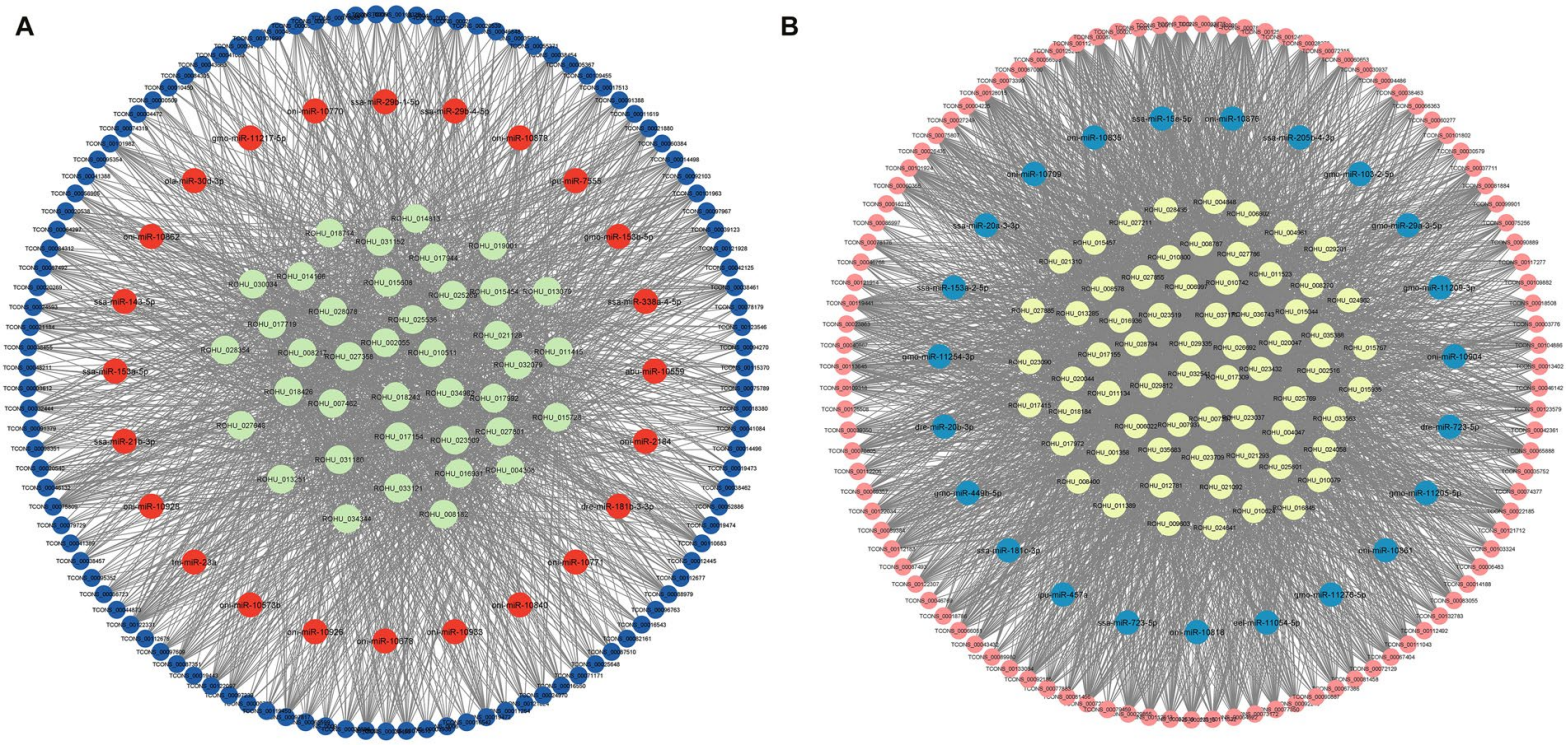
ceRNA (lncRNA-miRNA- mRNA) network of 8ppt salinity treated group. (**A**) The A* ceRNA network. Blue, orange, and green colour circles represent lncRNA, miRNA and mRNA respectively. Network includes 1746 edges, and 174 nodes consists of 103, 23 and 66 lncRNAs, miRNAs, and mRNAs respectively. (**B**) The B* ceRNA network. Pink, blue, and yellow colour circles represent lncRNA, miRNA and mRNA respectively. Network includes 3236 edges, and 192 nodes consists of 111, 24 and 39 lncRNAs, miRNAs and mRNAs respectively.

In 4ppt, A* network contains 163 lncRNA-miRNA-mRNA pairs, including 43 lncRNAs, 40 miRNAs, and 80 mRNAs. B* network includes 191 lncRNA-miRNA-mRNA pairs which include 53 lncRNAs, 60 miRNAs, and 78 mRNAs (Fig. 6 and Figshare Dataset 5 25). In 6ppt, A* network includes 103 lncRNA-miRNA-mRNA pairs which include 43 lncRNAs, 38 miRNAs, and 22 mRNAs. B* network contains 105 lncRNA-miRNA-mRNA pairs with 42 lncRNAs, 33 miRNAs, and 30 mRNAs (Fig. 7 and Figshare Dataset 5 25). In 8ppt, A* network contains 192 lncRNA-miRNA-mRNA pairs which include 103 lncRNAs, 23 miRNAs, and 66 mRNAs. B* network contains 174 lncRNA-miRNA-mRNA pairs which include 111 lncRNAs, 24 miRNAs, and 39 mRNAs (Fig. 8 and Figshare Dataset 5 25).

### Functional enrichment of the ceRNA network

**Fig. 9:**
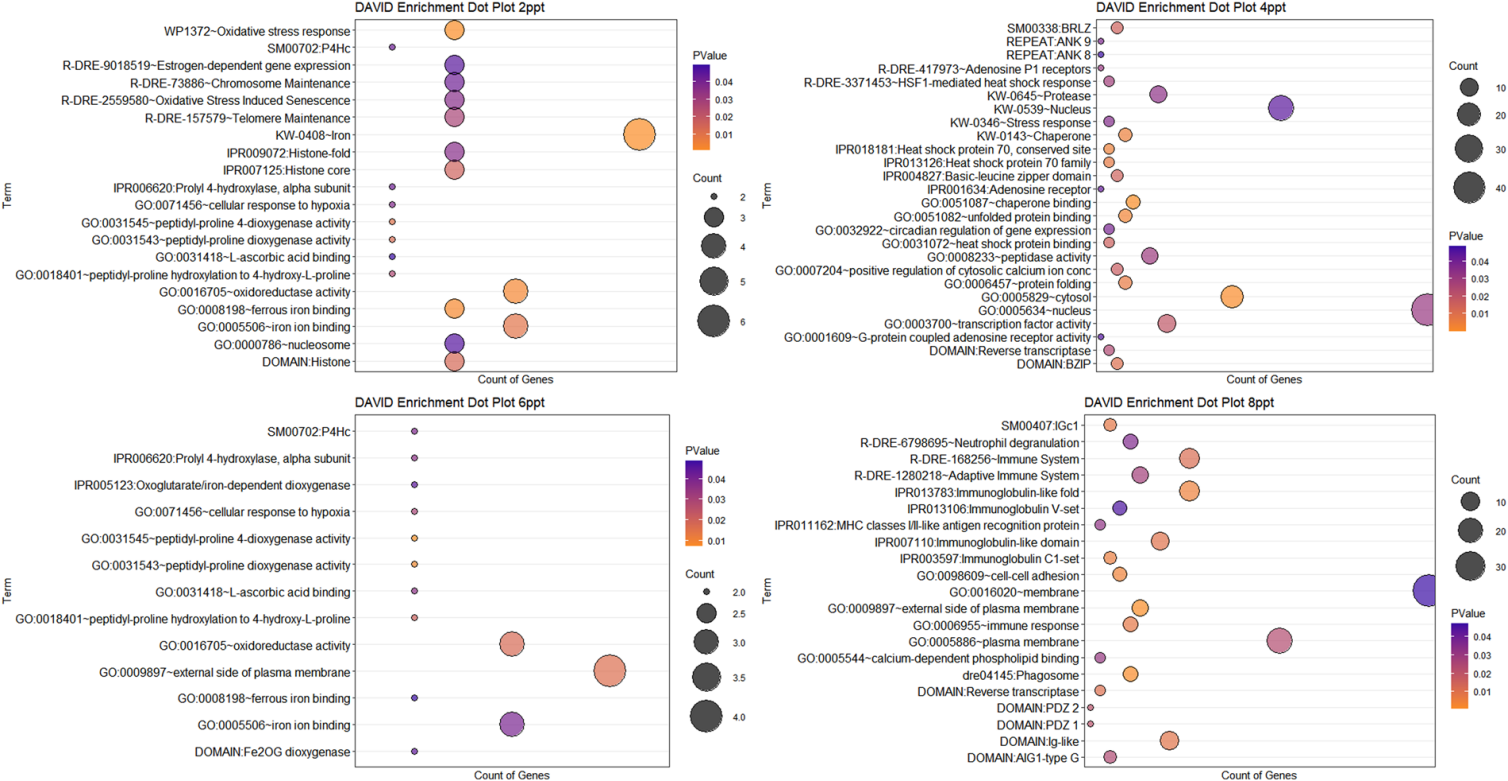
David enrichment plot for differentially expressed genes involved in ceRNA network for 2, 4, 6, and 8ppt treatment groups.

The functional enrichment and pathway analysis was performed using DAVID (https://david.ncifcrf.gov/). The significantly enriched terms classified in BP, CC, MF, and KEGG pathways were considered for identifying differentially expressed genes involved in salinity stress (Fig. 9 and Figshare Dataset 6^21^).

## Data Records

The raw FASTQ files were submitted to NCBI Sequence Read Archive https://identifiers.org/ncbi/insdc.sra:SRP384125 (2022)^26^. The files of differentially expressed mRNAs are published^18^. The tables representing the information of predicted putative lncRNAs and miRNAs, identified lncRNA-miRNA, miRNA-mRNA, lncRNA-miRNA pairs, lncRNA-miRNA-mRNA pairs, dataset used for RNA network and enrichment analysis are deposited on Figshare^21^. The *Labeo rohita* reference genome assembly and annotation used in this study are available on NCBI (https://www.ncbi.nlm.nih.gov/datasets/genome/GCA_004120215.1) (2019)^27^.

## Technical Validation

### RNA quality and integrity assessment

RNA quality was assessed using QIAxpert instrument (QIAGEN, Germany). A260/A280 ratio was ranged from 1.93–2.09, which is acceptable range. The quantity and integrity of the RNA were assessed with the Qubit 4 Fluorometer (Thermo Fisher Scientific, United States) and Agilent 2100 Bioanalyzer system (Agilent technologies, California, United States), respectively.

### RNA-seq data quality assessment

The raw fastq files were assessed for per base sequence quality, Phred sore, GC content and sequence duplication levels using FASTQC tool (v0.11.9).

## Data Availability

The following software’s and tools were used in this manuscript. No custom code was utilized during the analysis of the study. fastx toolkit (v0.0.14) https://github.com/agordon/fastx_toolkit segemehl (v0.2.0–418) http://legacy.bioinf.uni-leipzig.de/Software/segemehl STAR (v2.7.4a) https://github.com/alexdobin/STAR miRbase database https://www.mirbase.org miRanda (v3.3a) (http://www.microrna.org/microrna/home.do) FEELnc pipeline (v.0.2.1) https://github.com/tderrien/FEELnc CPC2 (v0.1) http://cpc2.gao-lab.org/ DESeq 2 (v.1.38.3) https://bioconductor.org/packages/release/bioc/html/DESeq2.html EdgeR (v.3.40.2) https://bioconductor.org/packages/release/bioc/html/edgeR.html featureCounts (v2.0.1) https://github.com/ShiLab-Bioinformatics/subread psych package (corr.test() for Pearson correlation coefficient) DAVID https://david.ncifcrf.gov/
